# Submucosal Oesophageal Haematoma Mimicking Acute Coronary Syndrome: A Case Report

**DOI:** 10.7759/cureus.91600

**Published:** 2025-09-04

**Authors:** Haider Hilal, Tanjit Singh, Okechukwu J Nkwocha, Tarek Alambrouk, Chinedu J Enwerem

**Affiliations:** 1 School of Medicine, St. George’s University, True Blue, GRD; 2 Medicine, Gateshead Health NHS Foundation Trust, Gateshead, GBR

**Keywords:** chest pain, endoscopy, hypertension, oesophageal haematoma, submucosal tear

## Abstract

Intramural oesophageal haematoma, also known as oesophageal apoplexy, is a rare condition characterised by a collection of blood within the submucosal layers of the oesophagus. Submucosal haematoma is a rare clinical entity that can occur spontaneously or secondary to trauma, toxin, medical intervention, or coagulopathy. Oesophageal haematoma develops due to multiple etiological factors, many of which were found to be due to impaired coagulation after myocardial infarction, thrombolytic treatment, vomiting, spontaneous in origin, or idiopathic. Oesophageal haematoma is classified as an acute oesophageal injury. It must be differentiated from other causes of acute chest pain, including myocardial infarction, aortic dissection, a Mallory-Weiss tear, and Boerhaave’s syndrome. CT and oesophagogastroscopy are the main modalities used to establish diagnosis. Following a case of spontaneous submucosal haematoma presenting with chest pain, it can be successfully managed conservatively. Prognosis is typically excellent when treated conservatively. We document the case of an 81-year-old male who presented with acute chest pain and was successfully treated with conservative management. The diagnosis turned out to be oesophageal submucosal haematoma. Along with the case report, we conducted a comprehensive review of case studies related to submucosal haematoma in the United Kingdom.

## Introduction

Injuries in the oesophageal wall are divided into a tear (Mallory-Weiss tear), a rupture (Boerhaave’s syndrome), or a rare presentation of haematoma (intramural, submucosal). Instrumentation in the oesophageal area may present as oesophageal haematoma, which can also occur spontaneously [1-4].

Sudden onset of chest pain is a usual presentation of submucosal haematoma of the oesophagus [2-5]. Submucosal or intramural haematoma of the oesophagus can occur spontaneously or following episodes of vomiting. Diagnosing submucosal haematoma is always a challenge in emergency settings due to its non-specific chest pain. This case report details a submucosal haematoma that was treated at Queen Elizabeth Hospital, Gateshead.

An extensive literature review was conducted on cases published since 1980, resulting in a total of 20 cases documented throughout the United Kingdom. The ages ranged from 42 to 80 years, with a mean age of 70.2 years. There were four males and 16 females for a male-to-female ratio of 1:4.

## Case presentation

An 81-year-old non-smoker male with a history of hypertension for the last 20 years presented with acute chest pain. His non-radiating chest pain episode lasted for 15 minutes, was relieved by sitting down, and was associated with retching and odynophagia. There was no history relating to haematemesis. He had a previous history of one transient ischemic attack (two years ago) and gastrointestinal (GI) surgery with an ileostomy 15 years ago. The patient’s medications included amlodipine 10 mg, ramipril 2.5 mg, and aspirin 75 mg.

On examination, our patient was afebrile and haemodynamically stable with a blood pressure of 140/75 mmHg, pulse of 78 beats/minute, and respiratory rate of 16 breaths/minute. His cardiac, respiratory, and abdominal examinations were unremarkable.

Blood test results showed haemoglobin of 124 g/L and platelet count of 215,000/µL. ECG showed sinus rhythm with no ischaemic changes. Troponin I level was normal initially, and in subsequent serial troponin I level testing, along with liver function tests and clotting factors. X-ray of the chest showed no pathology.

**Figure 1:**
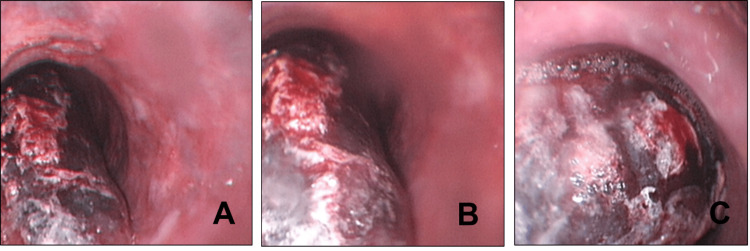
Upper gastrointestinal endoscopy showing necrotic mucosal Inflammation and submucosal haematoma in the upper-third of the oesophagus (A), mid-third of the oesophagus (B), and lower third of the oesophagus (C).

Upper GI endoscopy was performed and showed a submucosal haematoma in the lower half of his oesophagus, as shown in Figure 1. The patient was started on a proton-pump inhibitor (Omeprazole) infusion, and aspirin was discontinued.. Patient was booked for follow-up CT scans and endoscopic studies.

## Discussion

A thorough literature search was conducted on PubMed using keywords such as submucosal haematoma, chest pain, and oesophagus. The search resulted in various reported cases of oesophageal haematoma across the United Kingdom, which are summarised in Table 1.

**Table 1:** Reported cases of oesophageal haematoma across the United Kingdom.

Authors	Title	Year of publication	Age of the patient	Gender of the patient	Chief complaint	Location of the patient in the United Kingdom
Ambrose et al. [6]	A rare cause of haemetemesis and chest pain	2011	Elderly aged	Female	Haematemesis	London
Kerr [7]	Spontaneous intramural rupture and intramural haematoma of the oesophagus	1980	65	Female	Dysphagia and haematemesis	Norwich
			77	Female	Epigastric pain	
			80	Female	Chest pain and odynophagia	
			59	Female	Retrosternal/Epigastric pain	
			72	Female	Epigastric pain and haematemesis	
Herbertko et al. [8]	Spontaneous intramural haematoma of the oesophagus: appearance on computed tomography	1991	77	Female	Dysphagia and haematemesis	Southampton
			72	Female	Chest pain	
Modi et al. [9]	Dissecting intramural haematoma of the oesophagus	2005	42	Male	Chest pain and dysphagia	Plymouth
Thomasset and Berry [10]	Spontaneous intramural rupture and intramural haematoma of the oesophagus	2005	80	Female	Chest pain	Leicester
Alani [11]	Extensive esophageal haematoma with haematemesis treated by sclreosant injections	1995	79	Female	Chest pain and hematemesis	Blackburn
Huddy et al. [12]	Dissecting intramural haematoma of the oesophagus after thrombolysis for myocardial infarction	2005	67	Male	Odynophagia/Retrosternal pain	London
Ramnarine and Thorpe [13]	Spontaneous haematoma of the oesophagus	2004	50	Female	Superficial burning over the manubrium and retrosternal pain	Leeds
Disney et al. [14]	Intramural oesophageal hematoma: an unusual cause of acute chest pain	2014	79	Female	Central chest pain and Odynophagia	Dudley
Horan et al. [15]	Acute onset dysphagia associated with an intramural oesophageal haematoma in acquired haemophilia	2003	78	Male	Acute chest pain	Belfast
Cooray et al. [16]	Spontaneous intramural oesophageal haematoma in a patient with uncontrolled hypertension: an unusual chest pain aetiology	2017	69	Female	Chest pain	London
Lim and Everett [17]	Oesophageal hematoma and associated Mallory-Weiss tear	2004	80	Female	Chest pain	Leeds

Oesophageal haematoma is a rare diagnosis but has a significant clinical manifestation within a wider spectrum of oesophageal injuries such as Boerhaave’s syndrome and Mallory-Weiss tears. Unlike Boerhaave’s syndrome and Mallory-Weiss tears, which are associated with forceful vomiting, oesophageal haematoma pathogenesis often involves multiple factors that stem from various systemic and local insults to the oesophageal wall. While vomiting is a known trigger, oesophageal haematoma is frequently associated with alternative etiological factors. These include blunt or iatrogenic trauma, bleeding diatheses, and cardiovascular conditions such as aortic valve disease. Notably, a significant number of cases have been linked to impaired coagulation following thrombolytic therapy administered for myocardial infarction (MI), where the fragile vasculature of the oesophageal submucosa may become vulnerable to spontaneous haemorrhage. In some instances, the condition remains idiopathic, with no identifiable cause despite thorough investigation [18-20].

The diagnosis of oesophageal haematoma can be challenging due to its non-specific clinical presentation and its ability to mimic more critical thoracic emergencies such as MI or aortic dissection. The gold standard for diagnosis includes imaging studies such as a CT scan and an upper gastrointestinal endoscopy. On CT scans, contrast extravasation may be seen leaking into the pleural space, suggesting that there is a breach in the oesophageal wall, while endoscopy can directly visualise the submucosal haematoma, typically appearing as a bluish or purplish bulge within the oesophageal lumen [21-23]. Oesophageal haematoma was first documented by Kerr in 1980 in a 65-year-old female who presented with dysphagia and haematemesis symptoms. In the same case report, there were other patients of different ages presenting with a similar pattern of clinical manifestation, and they were all elderly females.

Generally, these cases demonstrate that oesophageal haematoma, even though it is a rare case, presents with varied clinical symptoms mimicking an acute chest syndrome such as aortic dissection or MI. The prevalence of chest pain and difficulty in swallowing among elderly female patients emphasises the importance of considering oesophageal haematoma as part of the differential diagnosis of atypical chest pain presentation, especially among elderly patients. Since the first documented case in 1980, a total of 20 cases have been reported across the United Kingdom. The age range of these patients is from 42 to 80 years, with a 70.2 years as mean age. There appears to be a marked gender disparity, with females being affected far more frequently than males, with a ratio of 4:1, respectively. This demographic trend suggests that elderly women may be particularly susceptible to developing this condition, possibly due to age-related mucosal atrophy and a higher incidence of anticoagulant use in this population.

Patients presented with a variety of symptoms. The most common complaints included odynophagia, acute retrosternal or epigastric chest pain, dysphagia, and haematemesis [24]. These symptoms often mimic cardiac or gastric pathology, complicating timely diagnosis. Interestingly, one UK case was reported to be asymptomatic, an atypical presentation that contrasts with the more dramatic symptomatology seen in most other cases. Despite its potentially alarming presentation, intramural haematoma of the oesophagus is typically managed conservatively. Treatment generally involves supportive care with PPIs, analgesics for pain control, and temporary cessation of oral intake if necessary. In most cases, the haematoma resolves spontaneously without the need for surgical intervention [24].

The cases of oesophageal haematoma across the United Kingdom, summarised in Table 1, spanned from 1980 to 2017 and encompassed a diverse range of patient demographics, clinical presentations, and locations. In terms of gender distribution, most patients were female, with 11 of the 14 cases involving women. Males accounted for only three cases. The age range of the affected individuals varied significantly, but most were elderly, commonly in their 70s or 80s. The oldest patient was 80 years old, and the youngest documented was 42 years old. Several entries listed multiple patients within one publication, including varied symptoms and ages. Clinical presentation varied widely. The most frequently reported symptom was chest pain, occurring either centrally or retrosternally. Chest pain was often associated with odynophagia and epigastric pain. A few cases presented with dysphagia, haematemesis, and superficial burning sensations over the chest area. One patient presented with an acute onset of dysphagia linked to an oesophageal haematoma in the context of acquired haemophilia. Another case was linked to MI thrombolysis treatment, highlighting oesophageal haematoma as a rare complication in such scenarios.

A unique case by Alani (1995) [11] involved a 79-year-old female who developed an extensive oesophageal haematoma because of sclerosant injections, suggesting a procedure-related aetiology. Another distinctive case described oesophageal haematoma in a 69-year-old female living with uncontrolled hypertension, indicating a possible link between cardiovascular risk factors and oesophageal vascular fragility [11]. Geographically, the cases were reported from a variety of locations across the United Kingdom; however, London has the highest number of cases, followed by Leeds. Other cities where oesophageal haematoma were reported include Belfast, Blackburn, Dudley, Leicester, Oxford, Plymouth, Norwich, and Southampton. Even though the condition is rare, it has been observed in various healthcare settings and regions across the United Kingdom.

## Conclusions

While oesophageal haematoma is rare, its diverse presentations and underlying causes necessitate high clinical suspicion, particularly in elderly patients with risk factors such as anticoagulation or recent chest trauma. Early recognition and non-invasive imaging are key to ensuring appropriate management and avoiding unnecessary surgical procedures.
